# Very Low Calorie Ketogenic Diet: What Effects on Lipid Metabolism?

**DOI:** 10.1007/s13668-024-00556-6

**Published:** 2024-07-15

**Authors:** Rosario Suarez, Sebastián Chapela, Natalia Daniela Llobera, Martha Montalván, Celina Andrade Vásquez, Andres Luciano Nicolas Martinuzzi, Christos S. Katsanos, Ludovica Verde, Evelyn Frias-Toral, Luigi Barrea, Giovanna Muscogiuri

**Affiliations:** 1 School of Medicine, Universidad Técnica Particular de Loja, Calle Paris, San Cayetano Alto, Loja 110107, Ecuador; 2 Facultad de Medicina, Departamento de Bioquímica Humana, Universidad de Buenos Aires, Ciudad Autónoma de Buenos Aires, Argentina; 3 Equipo de Soporte Nutricional, Hospital Británico de Buenos Aires, Ciudad Autónoma de Buenos Aires, Argentina; 4 Universidad Católica Santiago de Guayaquil, Av. Pdte. Carlos Julio Arosemena Tola, Guayaquil, 090615 Ecuador; 5 Fresenius Kabi, de Buenos Aires, Argentina; 6 Facultad de Medicina, Universidad Nacional del Comahue, Comahue, Argentina; 7 School of Life Sciences, Arizona State University, Tempe, AZ 85259 USA; 8 Department of Public Health, University of Naples Federico II, Via Sergio Pansini 5, 80131 Naples, Italy; 9 School of Medicine, Universidad Espíritu Santo – Samborondón, 0901952 Samborondón , Ecuador; 10 Department of Wellbeing, Nutrition and Sport, Pegaso Telematic University, Centro Direzionale Isola F2, Via Porzio, 80143 Naples, Italy; 11 Unità di Endocrinologia, Diabetologia e Andrologia, Dipartimento di Medicina Clinica e Chirurgia, Università degli Studi di Napoli Federico II, Via Sergio Pansini 5, 80131, Naples, Italy; 12 Centro Italiano per la cura e il Benessere del Paziente con Obesità (C.I.B.O), Unità di Endocrinologia, Diabetologia e Andrologia, Dipartimento di Medicina Clinica e Chirurgia, Università degli Studi di Napoli Federico II, Via Sergio Pansini 5, 80131 Naples, Italy; 13 Cattedra Unesco Educazione Alla Salute E Allo Sviluppo Sostenibile, University Federico II, 80131 Naples, Italy; 14 Facultad de Ciencias Médicas, Universidad de Guayaquil, Guayaquil, Ecuador

**Keywords:** Dyslipidemia, Hypercholesterolemia, Very-low-calorie ketogenic diet, Obesity, Low-density lipoprotein, Cholesterol, Lipid Metabolism

## Abstract

**Purpose of Review:**

This review aims to critically examine how VLCKD affects plasma lipoprotein, lipid and cholesterol metabolism. Cardiovascular disease is a worldwide health problem affecting millions of people and leading to high rates of mortality and morbidity. There is a well-established association between cardiovascular disease and circulating cholesterol. Various dietary recommendations are currently available for the management of dyslipidemia.

**Recent Findings:**

The very low-calorie ketogenic diet (VLCKD) is becoming increasingly popular as a treatment option for several pathological conditions, including dyslipidemia. In addition to being low in calories, the VLCKD's main feature is its unique calorie distribution, emphasizing a reduction in carbohydrate consumption in favor of fat as the primary calorie source. Lowering calorie intake through a VLCKD can reduce the endogenous production of cholesterol. However, if the foods consumed are from animal sources, dietary cholesterol intake may increase due to the higher fat content of animal products. When combined, these dietary practices may have opposing effects on plasma cholesterol levels.

**Summary:**

Studies investigating the impact of VLCKD on plasma cholesterol and low-density lipoprotein cholesterol levels report contradictory findings. While some studies found an increase in low-density lipoprotein cholesterol levels, others showed a decrease in total cholesterol and low-density lipoprotein cholesterol, along with an increase in high-density lipoprotein cholesterol.

## Introduction

Atherosclerotic cardiovascular disease (ASCVD) is a prominent contributor to cardiovascular disease (CVD), which is one of the primary contributors of morbidity and mortality in modern society [1]. In Europe, it claims the lives of approximately 2.2 million women and 1.8 million men every year [2]. However, males are more likely to die from CVD (490,000 vs. 193,000) before the age of 65 [1]. Also, even if patients have survived an initial CVD episode are more likely to experience another CVD incident [1, 2]. Prevention – which takes place as a coordinated effort at both the population and individual levels – is key to eliminating or greatly reducing the consequences of CVD and the associated disability [1]. A healthy lifestyle, including a proper diet and medication when necessary, are key strategies to reduce the incidence of ASCVD [1, 3].

Current guidelines for the management of hypercholesterolemia do not recommend following special dietary practices [1, 3]. Meanwhile, the European Society of Cardiology (ESC) and the European Atherosclerosis Society (EAS) state that a wide range of total fat consumption is acceptable. However, fat consumption corresponding to more than 35% of calories is usually associated with increased calorie and saturated fat intake. On the other hand, consuming lower amounts of fats and oils increases the risk of inadequate intakes of vitamin E and essential fatty acids, and may contribute to a decrease of HDL-C [1]. The majority of fat consumed need to be associated with n-6 and n-3 polyunsaturated fatty acids (PUFAs). Lowering the daily dietary cholesterol intake to 300 mg is recommended, especially in those with elevated plasma cholesterol levels [1, 4, 5].

The Very-low Calories Ketogenic Diet (VLCKD) consists of a nutritional protocol involving a reduction in daily carbohydrate intake and a relative increase in the percentages of calories consumed from protein and fat, where the daily calories consumed are < 800 [6]. Carbohydrates contribute ~13% of the total energy intake, fats ∼44%, and proteins ∼43% [7]. Recent research suggests that this diet, which was previously only recommended for patients with refractory seizures [8–12], may also be beneficial for patients with other pathologies, including impaired glucose control [13–15], obesity [16–20], polycystic ovary syndrome [9, 21–24] and cancer [25–28]. A VLCKD has also been studied as an addition to exercise regimes [18, 29–31]. Lowering plasma cholesterol levels in hypercholesterolemia via a VLCKD is based on the concept that consuming fewer calories will lead to a decrease in endogenous cholesterol synthesis. On the other hand, a diet high in fat content might increase the contribution of exogenous cholesterol, when the recommendation for dietary cholesterol is to consume no more than 300 mg per day [6].

This article reviews the benefits and drawbacks of VLCKD on plasma lipid and cholesterol metabolism in the context of common medical conditions. It will discuss the use, indications, and outcomes of VLCKD in various pathologies. In addition, it will focus on its use in patients with obesity, diabetes, and hypercholesterolemia, with specific emphasis on its effects on total plasma cholesterol, and the biology that underlies the observed outcomes.

## VLCKD in Chronic Diseases

Obesity is one of the major chronic diseases [20, 32–39], and VLCKD can be employed to induce weight loss. A meta-analysis published in 2020 of diets low in carbohydrates (< 40% of energy from carbohydrates) showed that these diets induced less weight loss compared with diets low in fat (< 30% of energy from fat) [40]. It has been hypothesized that suppression of hunger, which is a side effect of balanced, very low-energy diets, may be the cause of weight reduction induced by a VLCKD. Both diets result in ketosis, which is speculated to be behind the decrease in hunger resulting from a VLCKD. In addition, a ketogenic diet (KD) increases the energy expenditure [41]. Low-carbohydrate diets do not appear to be more effective in causing weight loss than other diets that restrict calories to the same extent such as low-fat vegetarian diets [42]. In 2021, a study by Hall et al*.* tested the effects of an animal-based KD and a plant-based, low-fat diet on appetite and weight loss [41]. This study reported levels of hunger and satisfaction that were comparable between groups. Also, both diets induced weight loss. However, the majority of the weight lost on the KD was associated with loss of fat-free mass [41]. These findings strengthen the argument that the initial, rapid weight loss resulting from a KD is mostly caused by the loss of fat-free mass [24, 32, 43, 44].

Regarding other chronic disease states, such as pediatric Type 1 Diabetes, a VLCKD may improve glycemia [32]. However, due to increased risk for malnutrition, failure to thrive, decreased bone density, hyperlipidemia, amenorrhea, and hypoglycemia, they are typically not utilized in this patient population. Both favorable and adverse health outcomes have been reported among individuals with type 1 diabetes [45]. A favorable health outcome of a KD in these patients is the improvement in blood glucose control. Nevertheless, this type of diet is associated with an increased frequency of hypoglycemic episodes. A review article strongly advised against the occurrence of long-term ketosis or hyperketonemia in those with type 1 diabetes [46]. It is important to keep in mind that patients with type 1 diabetes have higher rates of production of ketones along with decreased ketone clearance [46], which are linked to increased risk of microvascular, brain, kidney, and liver pathologies. Additionally, hyperketonemia in type 1 diabetes is linked to insulin resistance, non-alcoholic fatty liver disease, inflammation, and oxidative stress [32, 35, 47].

In Type 2 Diabetes, KD reduces appetite, promotes weight loss, lowers blood glucose, improves insulin sensitivity, and decreases HbA1c in the short term; those effects appear dependent on the amount of fat mass lost [40]. A study conducted in 2021 found that a plant-based diet had a higher glycemic load and, as it was expected, increased postprandial glucose and insulin levels more than a KD [48]. Finally, the benefits of VLCKD diets for type 2 diabetes are mostly due to weight loss, with these benefits tending to diminish over time [16].

In Non-alcoholic Fatty Liver Disease (NAFLD), hepatocytes with fat deposited inside them develop steatosis, which can lead to non-alcoholic steatohepatitis and ultimately raise the risk of hepatocellular cancer [32]. A common KD is characterized by an increase in the consumption of animal protein, cholesterol, and saturated fat, all of which are connected to insulin resistance, oxidative stress, and an elevated influx of free fatty acids into liver cells. Low-fat and VLCKD diets were investigated in numerous clinical trials involving overweight or obese people, and comparable reductions in intrahepatic fat were observed [49]. NAFLD is also likely to be exacerbated by diets that are low in dietary fiber and omega-3 fatty acids and high in simple carbohydrates, trans fats, and animal protein [50]. It has been proposed that entering a state of ketosis may help treat fatty liver [51], although the research that supports this claim is limited, and most of these studies also place restrictions on caloric intake, just like a VLCKD does [32].

Based on the so-called "Warburg effect," which occurs when cancer cells increase glucose consumption, upregulate glycolysis, and preferentially convert glucose to lactate, a number of publications recommended using KD for cancer patients [25, 52]. Thus, KD may stress cancer cells by nearly eliminating available glucose, at least in theory; however, few clinical trials have been conducted to test this hypothesis. In a 2018 systematic review of KD, no randomized clinical studies were identified for the management of gliomas, making it impossible for the authors to assess the effectiveness of KD for cancer survival [52]. A 2020 systematic review examined 13 research studies that used KD as an additional therapy to conventional cancer treatments [32]. The studies under consideration were modest in size, and the KD recommendations varied among studies. Both overall survival and progression-free survival showed inconsistent results [32]. Carefully designed, randomized clinical trials are required to ascertain the safety and efficacy of VLCKD in patients with cancer [53].

The utility of VLCKD in kidney disease is unclear. One potential risk for patients with chronic kidney disease (CKD) is the development of kidney stones [54]. The acidosis produced by KD may promote the production of stones by lowering the pH and citrate levels in the urine while raising the calcium levels. The onset of CKD is another potential concern for those without CKD consuming a KD. Although a "traditional" KD is not always high in protein, weight-loss diets that incorporate KD frequently result in high-protein intake (> 1.5 g/kg/d), and the acid load associated with this diet may exacerbate metabolic acidosis and renal impairments in patients with CKD [55].

**Fig. 1:**
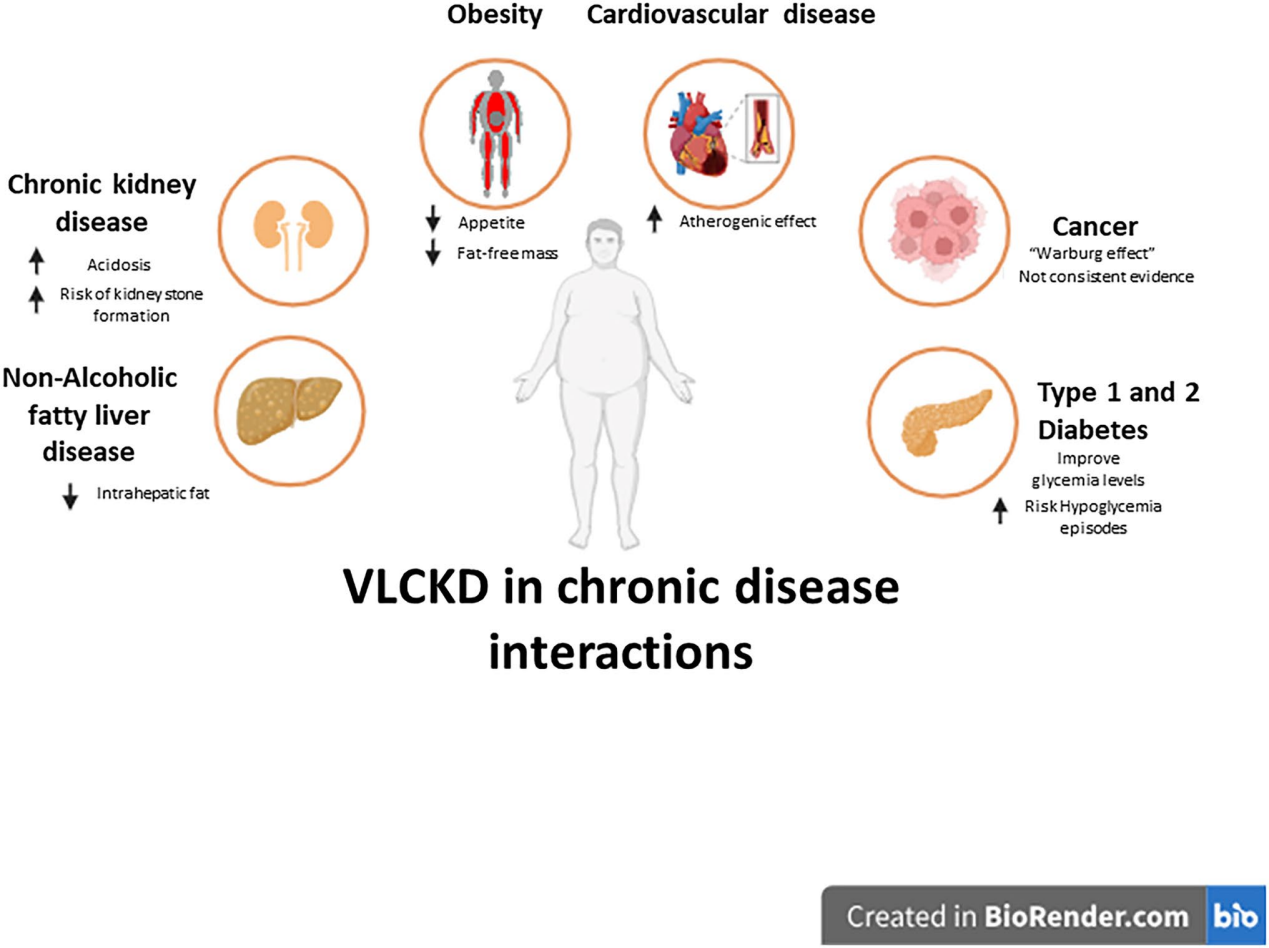
Disease states where VLCKD have been studied. Studies show decrease in fatty deposits in the liver in non-alcoholic liver disease. VLCKD induces weight loss and decreased appetite in obesity [6, 13, 40, 42, 57, 84, 85]. In type 1 diabetes, however, use of VLCKD is controversial due to the increase in ketone bodies that may be a common complication of the disease. In type 2 diabetes, findings are also controversial in regards to the regulation of Hb1Ac levels by a VLCKD [62, 64, 69, 70]. Finally, application of VLCKD in cancer and chronic kidney disease is still debated [27, 28, 52, 55]

A major concern in CVD is how low-carbohydrate diets affect plasma lipid levels is [40]. It is established that decreasing body weight lowers total cholesterol (TC). In a 2002 research study using the "Atkins diet", participants exhibited elevated LDL-C plasma levels, with an average increase of 18 mg/dL [56]. Comparable findings have been reported in a study by Yancy et al., in which 30% of participants had an LDL-C increase of over 10% [57]. On the other hand, patients following a conventional low-calorie diet saw an 11.1% decrease in Low-Density Lipoproteins Cholesterol (LDL-C) [57]. According to a 2018 study, subjects on VLCKD had an average 10% increase in LDL, which remained elevated over the course of a two-year follow-up [17]. However, a meta-analysis published in 2020 by Yuan et al. demonstrated that a VLCKD had no significant impact on LDL in people with type 2 diabetes [58]. These findings differ from those observed in healthy individuals, where fit adults on a KD exhibited on average a 3 kg weight loss but an increase in LDL-C by 35% [41]. Pathological conditions where a VLCKD have been studied are depicted in Fig. 1.

## Is VLCKD Recommended for Chronic Diseases? What About Hypercholesterolemia?

There are two sources of cholesterol: endogenous and exogenous. Endogenous cholesterol is synthesized in the liver in situations of excess energy where the Acetyl-CoA from glycolysis is directed in the synthesis of triglycerides and cholesterol [59, 60]. Triglycerides and cholesterol are transported by Very Low-Density Lipoproteins (VLDL) and subsequently converted to Intermediate Density Lipoproteins (IDL), which reach peripheral tissues after maturing into LDL [59, 60]. Exogenous cholesterol comes from the gastrointestinal tract, is transported via chylomicrons to the liver, and appears in the circulation into VLDL particles or chylomicrons [59, 60].

VLCKD diet has become very popular dietary approach in part due to findings from studies suggesting possible cardiovascular benefits. In a research published in 2002, 20 normolipidemic males of normal weight followed a ketogenic diet for six weeks to determine how it affected their postprandial and fasting blood biomarkers [56]. Subjects consumed a diet consisting of 61% fat, 30% protein, and 8% (or around 50 g/d) carbohydrates [56]. Fasting triglycerides (TG) (-33%), postprandial lipemia response to a high-fat meal (-29%), and fasting insulin concentrations (-34%) all decreased significantly in response to this KD [56]. LDL particle size increased significantly, but plasma concentrations of oxidized LDL remained unchanged [56]. HDL cholesterol increased significantly after three weeks on the KD [56]. When evaluating the overall cardiovascular risk, there were favorable responses in serum lipids, insulin, and subclasses of circulating lipids [56].

In a 2004 trial, 120 community volunteers with obesity and hyperlipidemia were selected at random, and all of them were advised to exercise in addition to receiving a low-fat or low-carbohydrate diet [57]. At 24 weeks, those on the low-carbohydrate diet had lower serum triglyceride concentrations than those on the low-fat diet (change, -0.84 mmol/L vs. -0.31 mmol/L [-74.2 mg/dL vs. -27.9 mg/dL]; P = 0.004) and higher concentrations of high-density lipoprotein cholesterol (HDL-C) (change, 0.14 mmol/L vs. -0. 04 mmol/L [5.5 mg/dL vs -1.6 mg/dL], P < 0.001) [57]. The change in LDL-C levels did not differ between the low-carbohydrate and low-fat diets (0.04 mmol/L [1.6 mg/dL] and -0.19 mmol/L [-7.4 mg/dL], P = 0.2) [57].

To determine if individuals on a VLCKD are able to regulate body weight and cardiovascular risk factors better in the long term compared to individuals on a conventional low-calorie diet, a meta-analysis of published studies was performed [61]. Findings revealed that over a follow-up period of at least 12 months, subjects assigned to the VLCKD exhibited greater increases in LDL-C and HDL-C levels and decreases in body weight, TG, and diastolic blood pressure (DBP) than those assigned to the traditional diet [61].

A study published in 2021 that contrasted a low-carbohydrate diet to a KD in type 2 diabetic mice showed that the KD increased lipid oxidation and decreased the de novo lipogenesis; however, the liver's lipid content increased [62]. In contrast, the low-carbohydrate diet decreased the concentrations of plasma triacylglycerols as well as markers of liver injury. Overall, these findings suggest that a low-carbohydrate diet may be advantageous in managing type 2 diabetes mellitus [62].

A meta-analysis of 10 randomized controlled trials (RCTs) published in 2021 found no statistically significant differences in HDL-C (d = 0.028, p = 0.934), LDL-C (d = 0.528, p = 0.173), or TG (d = -0.283, p = 0.222) in subjects with obesity when a balanced diet was compared to a KD [63]. According to these findings, a high-fat diet does not result in significant changes in body mass index (BMI), TC, HDL, LDL, or TG than a balanced diet [63]. It should be noted that trials with a higher percentage of females documented a decrease in plasma TGs. It is crucial to remember that this systematic review and meta-analysis report was based on a very limited sample of studies [63].

A meta-analysis of 21 RCTs published in 2022 reported how a KD affects cardiovascular disease (CVD) risk factors in patients with overweight or obesity [64]. The findings demonstrated that KD was more successful than control diets in reducing cardiovascular risk factors in overweight/obese patients, particularly in those with type 2 diabetes (i.e., significant decreases were documented for body weight, blood glucose, and lipid levels) [64]. A subgroup analysis focusing only on overweight/obese patients with type 2 diabetes revealed that plasma TG levels were significantly reduced in the KD group compared to the control-diet group (standardized mean difference (SMD), -0.32; p = 0.013), while the HDL levels showed a trend to increase with a p-value that tended to be significant (SMD, 0.07; p = 0,052) [64]. A subgroup analysis focusing on of overweight/obese subjects without type 2 diabetes revealed that HDL levels increased significantly more in the KD group compared to the non-KD (SMD, 0.13; p = 0.004) and that plasma TG levels showed a trend toward reduction and a P value that tended to be statistically significant (SMD, 0.15; p = 0.06). Overall, in comparison to non-KD, KD was generally more effective at lowering plasma TG (SMD, -0.2; p = 0.02) and raising the plasma HDL levels (SMD, 0.11; p = 0.03) [64]. However, the total cholesterol and LDL.C levels were not affected significantly by the KD [65]. A summary of the findings of these systematic reviews and meta-analyses reports is shown in Table 1.

**Table 1:** Studies involving KD and its effects on lipids and body composition

**Study type**	**Intervention**	**Outcome**	**Comment**
BUENO 2013 [61] Systematic review with meta-analysis n = 1577	VLCKD vs conventional low-fat diet	Body weight TG (mmol/l) HDL-C(mmol/l) LDL-C (mmol/l)	VLCKD-supported individuals achieved significantly greater long-term reductions in body weight, diastolic blood pressure, and TG, as well as greater increases in LDL and HDL compared with LFD-supported individuals.
LOPEZ ESPINOSA 2021 [63] Systematic review with meta-analysis Studies included in the review = 10	KD vs balanced diet	BMI TC TG (mmol/l) HDL-C(mmol/l) LDL-C (mmol/l)	When compared to balanced meals used to combat obesity, lipid profile data did not demonstrate any significant therapeutic benefit on BMI, TC, HDL-C, LDL-C or TG. It should be highlighted that BMI and TG dropped in the studies that included a higher percentage of female participants.
LUO 2022 [64] A meta-analysis of randomized controlled trials n = 1074	VLCKD vs non-ketogenic diets	L TC TG (mmol/l) (SMD, − 0.32; p = 0.013); No change in HDL-C(mmol/l); LDL-C (mmol/l) Lower	In obese/overweight T2DM patients and patients in general, VLCKD significantly reduced TG levels and increased HDL levels. They had no significant effect on changes in TC and LDL levels.

*KD* ketogenic diet, *BMI* body mass index, *TC* total cholesterol, *HDL* high-density lipoprotein, *LDL* low-density lipoprotein, *TG* triglycerides, *DM* diabetes mellitus, *LFD* low-fat diet. SOURCE: Self-made

Overall, current research shows that KD is beneficial in improving dyslipidemia, regulating insulin sensitivity, as well as protecting against CVD. Additional prospective studies are required to ascertain the long-term effects of KD on cardiovascular risk and their impact on cardiovascular events [64]. The effects of a KD on the lipid profile are not conclusive, so the effects of a KD on plasma lipids remain currently debatable.

## Side Effects of VLCKD in Hypercholesterolemia

VLCKD has been studied in various subject populations in regards to its effects on weight control/BMI and to rather lesser extent about its effects on plasma lipid profile. These studies include heterogeneous groups of subjects, whether they are patients with or without obesity and patients with or without diabetes mellitus. Although side effects of VLCKD have been described as mild and transient [64], their beneficial effects on reducing body weight and BMI have been confirmed in many studies. A meta-analysis reported by the European Guidelines for Obesity Management in Adults (EGOMA) evaluating the efficacy of VLCKD on body weight and body composition, glycemic and lipid profile in overweight/obesity participants found that VLCKD had a significant beneficial effect on body weight, fat mass, waist circumference, TC, triglyceridemia and insulin resistance, but reductions in glycemia, HbA1c, and LDL-cholesterol (LDL-C), were similar when compared to other weight loss interventions of similar duration; also, HDL cholesterol (HDL**-**C) did no change from baseline to follow-up in response to a VLCKD [6].

On the opposite side of experimental studies, two case reports have reported side effects with VLCKD regimes. A case report of a 56-year-old Hispanic woman who was placed on a KD for 30–40 days [66], showed that the patient's LDL-C and total cholesterol levels increased rapidly. Switching to a typical KD increases dietary cholesterol intake, causing total cholesterol and LDL-C to rise [66]. LDL subfractions were also modified, and in some cases, the predominant LDL subfractions in plasma were small and dense LDL particles [66]. The authors proposed that cholesterol mobilized from the adipose tissue as the fat cells shrink in response to a rapid weight loss due to the VLCKD may explain the increase in plasma LDL-C [66]. The other case report documented a myocardial infarction four weeks into the KD. According to the authors, oxidative stress, nutritional ketosis, and cardiac muscle degradation were the implicated factors, and given the absence of coronary atherothrombosis or elevated LDL-C [65], raising some concerns about the safety of KDs in the context of CVD. It is noted that, as it is described below, there are reports showing that a VLCKD can increase the plasma LDL-C [67].

Studies with diabetic mice on a KD or high-fat diet (HFD) have revealed that elevated PPAR-γ expression mediates cardiac dysfunction by upregulating certain mitochondrial enzymes [68]. This suggests that targeting PPAR-γ and its downstream mitochondrial enzymes offers a novel approach to preventing metabolic and myocardial dysfunction in diabetic patients. When diabetic mice were treated with KD versus a low-carbohydrate (LCH) diet, it was seen that KD, but not the LCH diet, promoted hepatic lipid accumulation despite increasing hepatic lipid oxidation and reducing de novo lipogenesis [69]. However, others have studied KD´s effects at the cellular and molecular levels in the context of reduced carbohydrate intake and serum insulin levels and reported increased insulin sensitivity and enhanced fat catabolism leading to reduced blood lipids [40, 70, 71]. It has also been suggested that a low-carbohydrate/high-fat KD significantly increases whole-body fatty acid oxidation and liver ketogenesis and reduces liver fat [72–74]. Furthermore, a KD raises LDL-C particle volume and size, which is expected to reduce the risk of CVD because it is smaller LDL particles that have higher atherogenic potential [75]. A KD also enhances the production of fibroblast growth factor-1 and promotes the hepatic clearance of TG [76]. Finally, a KD is shown to impact the production of endogenous cholesterol due to the reduction of serum insulin levels and because insulin activates hydroxymethylglutaryl-CoA reductase, an essential enzyme in cholesterol production, therefore preventing cholesterol biosynthesis [62, 77, 78].

Apart from the EGOMA meta-analysis already discussed above, several other studies have also reported on the modification of the lipid profile in response to VLCKD regimes (Table 2). In this regard, two studies conducted in different populations have reported significant improvements following a VLCKD in various metabolic parameters associated with the lipid profile [79, 80] Nevertheless, despite variable results on total, LDL-C, and HDL-C—likely due to differences in diet composition, genetic background, and physical activity of the groups—a systematic review from the Italian Society of Endocrinology found that randomized controlled trials (RCTs) involving weight-loss programs for obese individuals based on VLCKD reported greater improvements in plasma TG levels when compared to conventional diets. In most RCTs, total, LDL- and HDL-C were unchanged after 6 months on a VLCKD, while in other trials, HDL-C improved up to 12 months [7]. Another meta-analysis of 8 RCTs, reported improvements with a VLCKD in plasma TG levels in individuals with pre-diabetes or T2D, but no significant differences were found in any other variables [81].

**Table 2:** Summary of studies’ results regarding lipid profile

**Authors, year**	**Type of study**	**Subjects**	**Effect on TC**	**Effect on LDL-C**	**Effect on HDL-C**	**Effect on TG**
Alarim RA et al. 2020 [70]	Meta-Analysis	T2D patients		No change		
Caprio M et al. 2019 [7]	Systematic review and consensus statement	Obesity associated with different metabolic conditions (T2D, hypertension, dyslipidemia and others)	No change *	No change *	No change *	
Parry-Strong A et al. 2022 [81]	Meta-Analysis	T2D patients	No change *	No change *	No change *	
Muscogiuri G et al. 2021 [6]	Meta-Analysis	People with obesity		Similar to other interventions	No change	
Tragni E et al. 2021 [40]	Multi-center, prospective, uncontrolled trial	Women with overweight and obesity				
Vinciguerra F et al. 2023 [79]	Prospective	Post bariatric surgery subjects				
Rinaldi R et al. 2023 [80]	Prospective	People with overweight and obesity				
Basciani S et al. 2020 [82]	Randomized Pilot	People with obesity			No change *	
Ernesti I et al. 2023 [76]	Prospective before-after study	People with obesity			No change	
Barrea L et al. 2022 [83]	Prospective	People with obesity		No change		No change
Pandurevic S et al. 2023 [22]	Randomized controlled open label trial	Women with obesity and polycystic ovary syndrome	No change	No change	No change	No change
Tzenios N et al. 2022 [67]	Open-Label Pilot	Adults with Mildly Elevated LDL-Cholesterol				No change

*TC* total cholesterol, *LDL* low-density lipoprotein- cholesterol, *HDL* high-density lipoprotein- cholesterol, *TG* triglycerides. *not modified in most of the evaluated RCTs. SOURCE: Self-made

Two separate studies evaluating responses to VLCKD found a decrease in TC and LDL-C levels across patients, but no significant changes in HDL-C were reported [76, 82]. When compared to baseline, TC and HDL-C decreased but comparable responses were not evidence for LDL-C or TG levels in a prospective study [83]. These findings are in line with a case–control study that found that TC and HDL-C decreased significantly in the experimental group during the first eight weeks of the intervention; however, no significant differences were found in the lipid profile at the end of the study [22]. A pilot study with 14 healthy adults with mildly elevated LDL-C reported that following a VLCKD, LDL-C, and HDL-C increased with no significant changes in plasma TG. Nonetheless, the latter findings are considered of minimal clinical relevance since all study participants had a lipid profile associated with a non-atherogenic phenotype A (high HDL-C and low TG/HDL-C ratio) [67].

In summary, although many meta-analyses have reported improvements in plasma TC levels with VLCKD, responses associated with other components of the lipid profile have been quite variable, with a trend for neutral effects when these were evaluated over the long term. Discussing these findings and how they might be interpreted in relation to earlier studies and working hypotheses is essential. It is crucial to examine the findings and their implications from as wide an angle as possible and highlight potential future research directions.

## Conclusions

Studies demonstrating the advantages or disadvantages of the KD have increased in number due to the growing popularity of these diets. The diet’s theory of lowering cholesterol levels stems from consuming less calories, thus reducing endogenous cholesterol production. On the other hand, exogenous cholesterol—which is recommended to be less than 300 mg per day—can contribute more when dietary calories are mostly associated with fats.

Currently, there is conflicting evidence in that some studies show improvements in total cholesterol and LDL-C levels, while others report opposite results. Also, the populations studied have been rather diverse. These studies have included non-diabetic patients with obesity, diabetic patients, and largely dyslipidemic patients. Also, there were differences in the diets followed by the non-VLCKD groups compared with the VLCKD groups. Therefore, future studies need to be carried out with well-defined populations, and treating physicians should closely supervise and use caution while recommending this diet.
